# Matrix metalloproteinase-9 (MMP-9) Expression in Non-Small Cell Lung Carcinoma and Its Association with Clinicopathologic Factors

**DOI:** 10.30699/ijp.2020.95177.1940

**Published:** 2020-07-16

**Authors:** Amir Hossein Jafarian, Melika Kooshki forooshani, Hossein Reisi, Nema Mohamadian Roshan

**Affiliations:** Pathology Department, School of Medicine, Mashhad University of Medical Sciences, Mashhad, Iran

**Keywords:** Immunohistochemistry, Matrix metalloproteinase 9, Non-small-cell lung carcinoma

## Abstract

**Background & Objective:**

Matrix metalloproteinases-9 (MMP-9) is one of the most important enzymes to breakdown extracellular matrix which plays a major role in tumor invasion and metastasis. This study aimed to determine tumor MMP-9 expression in non-small-cell lung carcinoma (NSCLC) and whether it is associated with histopathologic factors and has prognostic value to affect overall survival (OS).

**Methods:**

The specimens of 92 patients with NSCLC diagnosis were included. Tumor sections were stained by immunohistochemistry method. Using scores for the percentage of cells positively stained and the intensity of staining, MMP-9 expression total score was classified as low-score (scores of 0 to 2), moderate-score (scores of 3 to 5), or high-score (scores of 6 or 7). OS was defined as the time interval since the diagnosis of NSCLC to the status at the last follow-up (dead or alive). The follow up period was up to 70 months.

**Results:**

About 74% of undifferentiated specimens (grade III tumors) showed high scores for MMP-9 expression which was significantly higher than moderately differentiated tumors (25% had high scores for MMP-9 expression) and well differentiated ones which did not have high scores (*P*<0.001). A total of 74 patients (80.4%) died during the follow-up period. Of this, 36% had high scores for MMP-9 expression. In contrast, none of the patients who were alive at the last follow-up had high scores for MMP-9 expression (*P*<0.001). Median OS was significantly lower in high score group (6 months) compared to moderate score (9 months) and high score group (15 months) (*P*<0.001).

**Conclusion:**

MMP-9 expression may serve as a significant prognostic factor for mortality and overall survival in NSCLC. Undifferentiated tumors significantly express higher MMP-9 immunohistochemically.

## Introduction

Lung cancer is considered as the most lethal malignancy worldwide and is the second most common malignancy in both males and females. In fact, lung cancer accounted for about one-quarter of cancer-related deaths (158,040 deaths) in the US in 2015 (1) and 1,380,000 deaths globally in 2008 (2). Lung cancer (bronchogenic carcinoma) is broadly divided to small cell lung cancer (SCLC) or non-small-cell lung carcinoma (NSCLC). NSCLC constitutes more than two-thirds of lung cancers and is divided into three main subtypes, namely squamous cell carcinoma (SCC), adenocarcinoma, and large cell carcinoma. The 5-year survival rate even after resection of early-stage NSCLC is about 50-60% (3,4). One of the major components in determining prognosis of NSCLC is metastasis, which comprises a major role in tumor node metastasis (TNM) staging system in determining the prognosis of NSCLC and help in its management.

Metastasis of NSCLC involves several complex pathologic processes. An important one is dynamic interaction that occurs between tumoral cells, basement membrane, and extracellular matrix (ECM) (5). Invasion of the ECM and loss of tissue homeostasis is one of the important mechanisms through which metastasis can occur. In other words, tumoral cells break through collagenous barriers of the basement membrane to invade the ECM (6,7). One of the endopeptidases that degrade ECM, during angiogenesis, via its enzymatic activation is the family of matrix metalloproteinases (MMPs) (8). This family of endopeptidases includes 24 endopeptidases that destruct ECM (9). 

Perhaps, the most important and widely studied component is MMP-9, known as gelatinase B. MMP-9 has been shown to represent the invasiveness of NSCLC, through induction by vascular endothelial growth factor receptor-1 (VEGFR-1)/Flt-1 tyrosine kinase (10). VEGF is known to affect vascular endothelial cells and having anti-apoptotic properties (8). MMP-9 is the most studied and important MMP family member in cancer tissue remodeling via degradation of denatured collagens (gelatins) of ECM, in particular collagen types V, VII, IX, as well as elastin and fibrin among others (9,11). 

Considering the mentioned evidence studies have been done to determine over expression of MMP-9 and its relationship with prognosis and survival of NSCLC patients (6,12). For instance, a previous study showed that MMP-9 expression, observed in 38.6% of resected NSCLC specimens, was significantly correlated with poor survival and recurrence only among adenocarcinoma tumors, but not in SCC group (13). Another study showed higher rate of MMP-9 expression in NSCLC in 61% of specimens (12). In contrast, some studies have mentioned MMP-9 expression as a positive prognostic factor in NSCLC (14). Even, experts mentioned that MMP-9 inhibition by PinX1 (PIN2/TRF1-interacting telomerase inhibitor 1) may act as a metastatic tumor suppressor in breast cancer (15). Such observations have made MMPs as potential targets for therapeutic approaches, especially in advanced NSCLC where operation and resection of the tumor becomes less promising. 

Incidence of malignancy, overall, is raising in developing countries due to population aging and cancer-related habits namely smoking, physical inactivity, obesity and stress (16). Evidence shows in the near future, the incidence of lung cancer is unlikely to reduce but the burden will shift from the developed to the less-developed countries (16) including Iran. Hence, despite a number of studies on similar issue, we decided to determine tumor MMP-9 expression in Iranian patients with NSCLC which has not been studied yet and also investigate MMP-9 expression prognostic value for overall survival as well as its association with demographic, clinical, and histopathologic factors. 

##  Materials and Methods

**Setting and Samples**

This cohort study was done in our academic medical center in a 10-year period from 2006 to 2016. During the study period, 92 patients who had received the diagnosis of NSCLC (adenocarcinoma, SCC, and large cell carcinoma) were included. They had undergone bronchoscopy or total surgical resection of the mass for lung tumors. 

**Data Collection**

The clinical variables were collected via reviewing medical records and contacting patients for survival data. The variables documented were age, gender, resectable tumors, chemotherapy, and OS (time interval from the diagnosis of NSCLC to the last follow-up which defined as alive or dead). 

**Immunohistochemical Staining**

Formalin-fixed and paraffin-embedded specimens were retrieved from the archive of Pathology Department of our medical center (Ghaem hospital, Mashhad) and were reviewed and those with sufficient tissue and stroma were selected. The specimens were examined by two pathologists to re-confirm the diagnosis and grade of the tumors and the appropriate place for immunohistochemistry was selected. The paraffin-embedded tissues were sectioned (3- to 4-micrometer thickness sections) by a microtome. Then, the sections were fixed at 80ºC for 45 minutes and then they were deparaffinized using xylol for 5 minutes. Hydration was done by 100% alcohol, 90% alcohol, 80% alcohol, and 70% alcohol (each for 5 minutes). Then, the slides were placed at 94-98ºC in phosphate-buffered saline for 30 minutes. The slides were treated by the primary MMP-9 antibody (ready-to-use rabbit monoclonal anti-MMP-9, Biogenex, USA) without dilution and refrigerated overnight at 4ºC and then was washed in Tris-buffered saline (TBS) for 5 minutes. Then, the slides were exposed to horseradish peroxidase conjugated secondary antibody (EnVision, Dako A/S, Glostrup, Denmark) for 30 minutes at room temperature and then were washed in TBS for 5 minutes. Staining was performed with hematoxylin for several seconds and then washing was done. Then, using xylol and 70% alcohol, dehydration was done and the slides were examined by Nikon microscope (×100 and ×400). 

**MMP-9 Expression Score**

The slides were examined by a pathologist who was blinded to the clinical variables and survival of the patients using Nikon microscope. The percentage of cells that was positively stained was classified as <10% (score= 0), 10-25% (score= 1), 26-50% (score= 2), 51-75% (score= 3), and 76-100% (score= 4). The staining intensity was categorized as negative, weak, moderate, and strong and respectively scored as 0, 1, 2, and 3. The scores assigned to percentage of cells and staining intensity were added and the resulted score was MMP-9 expression score. According to this final score, the specimens were classified as low-score (scores of 0 to 2), moderate-score (scores of 3 to 5), and high-score (scores of 6 or 7) MMP-9 tumors. Non-neoplastic peripheral tissue of the lung tumor specimen from the same patient was regarded as the control group (17).

**Statistical Analyses**

Descriptive indices including frequency, percentage, mean and its standard deviation (±SD) were used to express data. The Wilcoxon test was used to compare the percentage of cells positively stained as well as the intensity of staining between tumor group and control group. The Chi-squared test was used to compare the frequency of the studied variables among three groups of specimens with low, moderate, and high score for MMP-9 expression. The Kaplan-Meier analysis was used to compare the overall survival among three groups of specimens (low, moderate, and high scores for MMP-9 expression). A P-value of less than 0.05 was considered as statistically significant. All analyses were done using SPSS 18.0 (SPSS Inc., Chicago, Ill., USA). 

*Ethics*

The study protocol was approved by the Research Deputy of Mashhad University of Medical Sciences (MUMS). No intervention was performed here and patient informed consent was not considered to be required. 

## Results

**Clinical and Histopathologic Variables**

There were 68 males (73.9%) and 24 females (26.1%). Mean (±SD) age of the sample was 60.28 (±10.39) years. There were 44 adenocarcinoma (47.8%), 44 SCC (47.8%), and four patients (4.3%) with large cell carcinoma. Grades I (well-differentiated), II (moderately differentiated), and III (undifferentiated) tumors were diagnosed respectively in 30 (32.6%), 39 (42.4%), and 23 (25%) patients. Thirty-nine specimens were moderately differentiated (grade II). Surgical resection had been done for 37 patients (40.2%) and chemotherapy had been administered for 68 cases (73.9%). A total of 74 patients died during the follow-up period (1 month to 70 months). 

**Immunohistochemistry Findings**

Table 1 summarizes the comparison of percentage of positively stained cells, staining intensity, and the total score of MMP-9 expression between NSCLC and control group. Eighteen patients (19.6%) had scores of 0 for intensity of staining. In 76 specimens (82.6%), MMP-9 expression was more than 1%. MMP-9 was expressed in normal lung tissue, but its expression score was significantly higher in tumor group. Likewise, this difference was seen regarding staining intensity and the total score for MMP-9 expression. 

**Table 1:** Comparison of percentage of positively stained cells for MMP-9, staining intensity, and the total score of MMP-9 expression between non-small cell lung cancer (NSCLC) and control groups

		NSCLC (N= 92)	Control (N= 92)	P-value
Percentage of positively stained cells	< 1%	18 (19.6%)	18 (19.6%)	< 0.001
	1 to 25%	24 (26.1%)	74 (80.4%)	
	26 to 50%	12 (13%)	0	
	51 to 75%	27 (29.3%)	0	
	76 to 100%	11 (12%)	0	
Staining intensity	No staining	18 (19.6%)	18 (19.6%)	< 0.001
	Weak	28 (30.4%)	74 (80.4%)	
	Moderate	19 (20.7%)	0	
	Strong	27 (29.3%)	0	
Total score for MMP-9 expression	Low (0-2)	42 (45.7%)	92 (100%)	< 0.001
	Moderate (3-5)	23 (25%)	0	
	High (6 or 7)	27 (29.3%)	0	

* P-value <0.05

**Table 2:** Association of MMP-9 expression (according to total score of MMP-9 expression) in non-small cell lung cancer (NSCLC) with the studied variables^1^ among 92 patients

			Low-score	Moderate-score	High-score	Sig.
Gender, male			28 (41.2%)	19 (27.9%)	21 (30.9%)	0.15
Age, years	> 60		22 (48.9%)	6 (13.3%)	17 937.8%)	0.24
	≤ 60		20 (42.6%)	17 (36.2%)	10 (21.3%)	
Tumor grade	I		28 (93.3%)	2 (6.7%)	0	< 0.001
	II		10 (25.6%)	19 (48.7%)	10 (25.6%)	
	III		4 (17.4%)	2 (8.7%)	17 (73.9%)	
Histologic cell type	ADC		18 (40.9%)	13 (29.5%)	13 (29.5%)	0.05
	SCC		24 (54.5%)	9 (20.5%)	11 (25%)	
	Large		0	1 (25%)	3 (75%)	
Surgical resection^2^			18 (48.6%)	8 (21.6%)	11 (29.7%)	0.4
Chemotherapy^3^			30 (44.1%)	19 (27.9%)	19 (27.9%)	0.77
Overall survival		Dead	29 (32.2%)	18 (24.3%)	27 (36.5%)	0.001
		Live	13 (72.2%)	5 (27.8%)	0	

**Table 3:** Overall survival comparison among three groups of non-small-cell lung carcinoma based on MMP-9 expression (Kaplan-Meier analysis)

	Median overall survival, month	95% confidence interval	P-value
Low score (0 to 2)	15	11.44 to 18.56	< 0.001
Moderate score (3 to 5)	9	7.15 to 10.84	
High score (6 to 7)	6	3.45 to 8.54	

**Association of MMP-9 Expression with the Studied Variables**

**Fig. 1:**
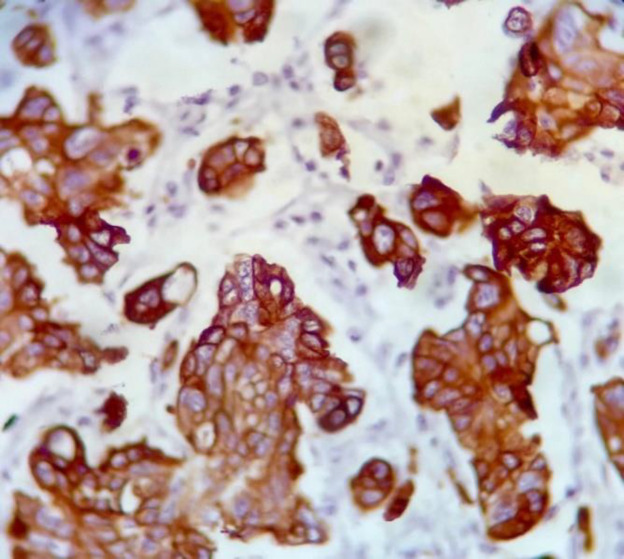
MMP-9 strongly positive in moderately differentiated lung adenocarcinoma. ×400

**Fig. 2:**
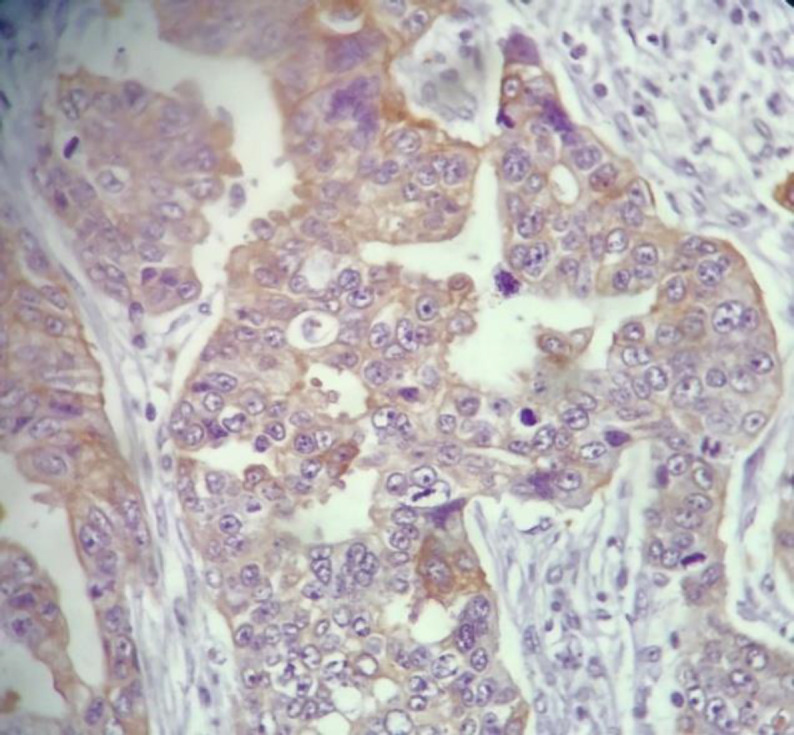
MMP-9 weak positivity in moderately differentiated lung adenocarcinoma. ×400

**Fig. 3:**
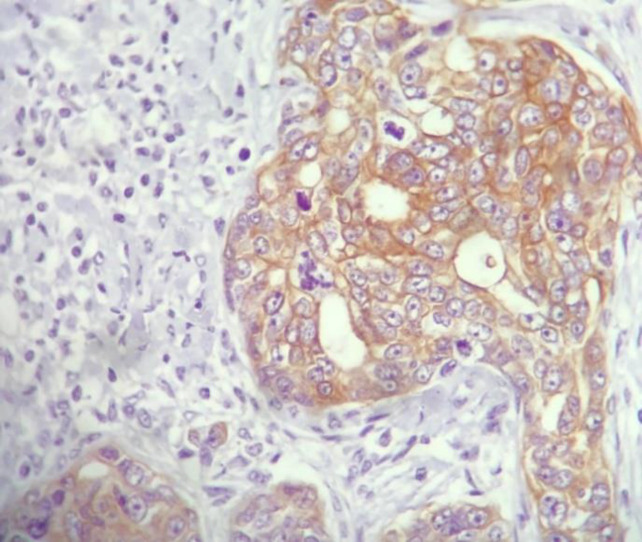
MMP-9 moderate positivity in moderately differentiated lung adenocarcinoma. ×400

Table 2 shows association of MMP-9 expression with the studied variables. As observed, no significant association was found between MMP-9 expression and gender, age (considering the median value of 60 years), surgical resection, and chemotherapy. However, 73.9% of undifferentiated specimens (grade III tumors) showed high scores for MMP-9 expression which was significantly higher than moderately differentiated tumors (25% had high scores for MMP-9 expression) and well differentiated ones which did not have high scores (*P*<0.001) (Figures 1, 2, 3). Also, of 74 patients who died during the observation period, 36% had high scores for MMP-9 expression. In contrast, none of the patients who were alive at the last follow-up had high scores (*P*<0.001). With regard to histologic cell type, the difference was not statistically significant, though it was marginally significant (*P*=0.05). This may be due to the low sample size of large cell carcinoma (4 patients) of whom three patients (65%) had high scores for MMP-9 expression. The distribution of adenocarcinoma and SCC were even in MMP-9 expression score groups.

**Fig. 4:**
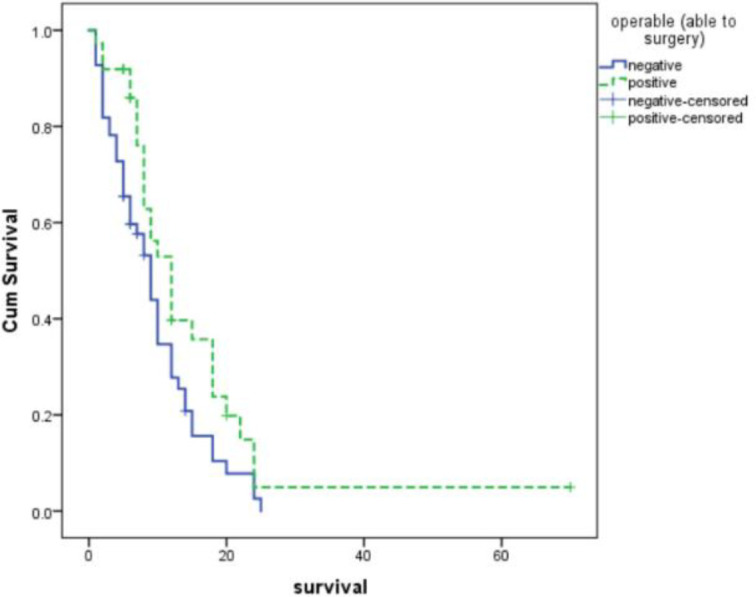
Overall survival curves for three groups of non-small-cell lung carcinoma specimens based on MMP-9 expression score

**Fig. 5:**
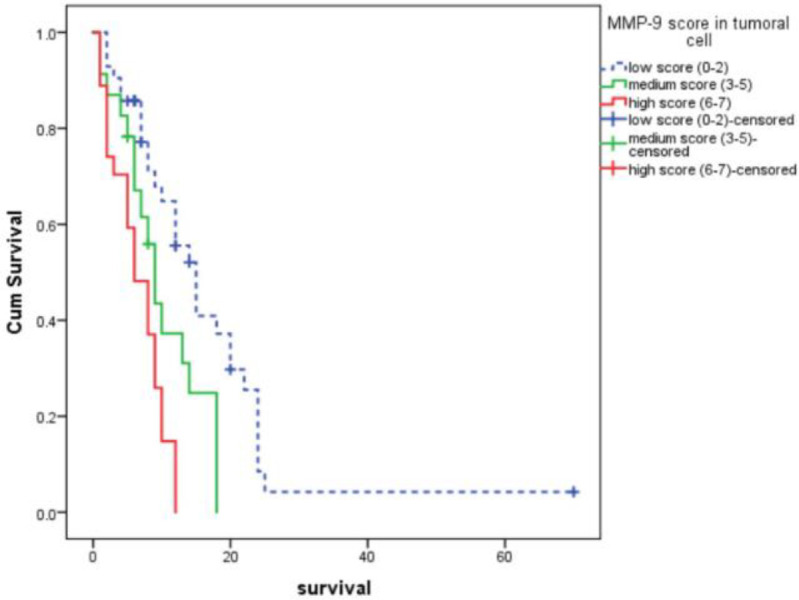
Overall survival curves for two groups of non-small-cell lung carcinoma specimens based on operable or non-operable lung tumor

**Association of MMP-9 Expression with Survival**

Median overall survival was significantly higher in low-score group compared to moderate and high score MMP-9 expression groups. With increased score of MMP-9 expression, the overall survival decreased significantly (Table 3) (Figure 4). 

Association of MMP-9 Expression with Surgical Resection of the NSCLC

Median overall survival time was significantly higher in operable group (12 months, 95% CI=8.86 to 15.13 months) compared with not operable group (9 months, 95% CI= 6.96 to 11.03 months) (*P*=0.03) (Figure 5). 

## Discussion

Based on the obtained findings, high score for MMP-9 expression was a significant prognostic value for mortality and overall survival in NSCLC patients. Patients whose tumors were diagnosed as undifferentiated also had significantly higher MMP-9 expression scores. Several studies have addressed the role of MMPs in the pathogenesis of malignancies, including NSCLC. MMPs have a role in proteolytic degradation of ECM and promote tumor cell migration and metastasis (18). They also promote tumor vascularization and silencing of MMP-9 expression has been noted to result in a less aggressive phenotype (19). Here, we observed that MMP-9 was expressed in both malignant cells and normal lung tissue. However, more than half of malignant specimens had MMP-9 expression of more than 25%. While none of the normal lung tissue samples showed MMP-9 expression of > 25%. It has been shown that MMP-9 is expressed not only in lung tumor, but also in healthy lung tissues. However, its expression was significantly higher only in adenocarcinoma lung cancer (20). This observation has also been reported in another study which showed MMP-9 expression being significantly higher in both NSCLC and small-cell lung cancer (SCLC) compared to normal lung tissues of the same patients. Even tissue and serum MMP-9 activity was higher in NSCLC compared to SCLC (21).

We did not analyze MMP-9 expression distinctly in adenocarcinoma and SCC subgroups. However, there is evidence that prognostic value of MMP-9 may be of more importance in adenocarcinoma and not in SCC. In a previous study including 417 patients with NSCLC, tumoral cell MMP-9 expression was observed in 38% of specimens (13). The authors observed that MMP-9 was a poor prognostic factor only for adenocarcinoma, not SCC. MMP-9 was significantly associated with both disease-free survival and OS just in adenocarcinoma group. Some experts believe that tumor histology distinction should be considered when interpreting MMP-9 over expression (22). This difference may be somehow related to the definition of MMP-9 positivity in the mentioned study (13). They multiplied percentage of positively stained cells and staining intensity and a cut-off value of 10 (possible values ranged from 0 to 300) was used to describe MMP-9 expression. Hence, only 36% of the samples were designated as having MMP-9 expression. Various studies have used various methods to describe MMP-9 expression and a widely accepted scoring system is not yet available. 

We observed that a higher number of patients with undifferentiated tumors had higher scores for MMP-9 expression. This is compatible with previous studies that showed MMP-9 expression associated with higher grade tumors as well as lymph node involvement (21,22). MMP-9 expression was an indicator for poor prognosis and OS. This has been reported in several studies earlier (6,13,23,24,25). All the studies reported that MMP-9 expression was a poor prognostic factor for 3-year or 5-year survival independently among other histopathologic or clinical factors. Most studies, similar to ours, have used immunohistochemistry to determine MMP-9 over expression. In addition to tissue MMP-9, some studies have studied serum MMP-9 activity and reported that serum MMP-9 was also a prognostic factor in NSCLC (21). 

Some studies have tried to find other molecular pathway related to MMP9 expression in the course of disease progression. For example is it suggested that CTHRC1 which is directly correlated with MMP7 and MMP9 expression, promotes NSCLC invasion, lymphatic metastasis, distant metastasis by up-regulating MMP7 and MMP9.(26,27)

In other sort of studies researchers tried to find out MMP-9 activity in various lung diseases. For instance, MMP-9 serum level was found to be increased by ~1.6 folds (*P*<0.05) in lung cancer patients compared to healthy donors but in comparison with COPD patients, there was insignificant change in the serum levels of MMP-9 and MMP-2, however, it is reported that a selective inhibitor of MMP-9 and -12 decreases the inflammatory process correlated with exposure to cigarette smoke in COPD patients (28,29).

Although MMP-9 has been found to be a potential cancer biomarker in different types of malignancies, discovering its exact translational application value (alone or as one biomarker within a biomarker combination) in one specific cancer, still needed systematic high quality evaluations (30).

## Conclusion

Immunohistochemical MMP-9 expression in NSCLC (both adenocarcinoma and SCC) could be a significant prognostic factor. Patients with higher scores of MMP-9 expression may have significantly lower OS. MMP-9 expression is also significantly associated with tumor grade. Undifferentiated tumors (grade III) may have higher scores of MMP-9 expressions. MMP-9 expression in NSCLC could be helpful to predict prognosis and evaluate the tumor grades. This can be helpful in tailoring treatment. More aggressive treatment can be performed in patients with higher scores of MMP-9 expression. 
